# Defining the architecture of KPC-2 Carbapenemase: identifying allosteric networks to fight antibiotics resistance

**DOI:** 10.1038/s41598-018-31176-0

**Published:** 2018-08-27

**Authors:** Ioannis Galdadas, Silvia Lovera, Guillermo Pérez-Hernández, Melissa D. Barnes, Jess Healy, Hamidreza Afsharikho, Neil Woodford, Robert A. Bonomo, Francesco L. Gervasio, Shozeb Haider

**Affiliations:** 1 Department of Chemistry, University College London, London, UK; 2 UCB BioPharma, Discovery Chemistry, New Medicines, UCB Pharma, B-1420 Braine-l’Alleud, Belgium; 3 Department of Mathematics and Computer Science, Freie Universitat Berlin, Berlin, Germany; 4 Department of Medicine, Case Western Reserve University & Research Service, Louis Stokes Cleveland Department of Veterans Affairs, Cleveland, Ohio USA; 5 Institute of Structural and Molecular Biology, University College London, London, UK; 6 Antimicrobial Resistance and Healthcare Associated Infections (AMRHAI) Reference Unit, National Infection Service, Public Health England, London, UK; 7 UCL School of Pharmacy, University College London, London, UK

## Abstract

The rise of multi-drug resistance in bacterial pathogens is one of the grand challenges facing medical science. A major concern is the speed of development of *β*-lactamase-mediated resistance in Gram-negative species, thus putting at risk the efficacy of the most recently approved antibiotics and inhibitors, including carbapenems and avibactam, respectively. New strategies to overcome resistance are urgently required, which will ultimately be facilitated by a deeper understanding of the mechanisms that regulate the function of *β*-lactamases such as the *Klebsiella Pneumoniae* carbapenemases (KPCs). Using enhanced sampling computational methods together with site-directed mutagenesis, we report the identification of two “hydrophobic networks” in the KPC-2 enzyme, the integrity of which has been found to be essential for protein stability and corresponding resistance. Present throughout the structure, these networks are responsible for the structural integrity and allosteric signaling. Disruption of the networks leads to a loss of the KPC-2 mediated resistance phenotype, resulting in restored susceptibility to different classes of *β*-lactam antibiotics including carbapenems and cephalosporins. The ”hydrophobic networks” were found to be highly conserved among class-A *β*-lactamases, which implies their suitability for exploitation as a potential target for therapeutic intervention.

## Introduction

Bacterial multi-drug resistance has become a major public health concern and a grand challenge for researchers. Carbapenems, in particular, are considered antibiotics of last resort in the treatment of serious nosocomial Gram-negative infections^1–3^. Hence, acquired carbapenem resistance among Gram-negative bacteria has repeatedly been highlighted as one of our most urgent clinical and public health threats, especially when mediated by carbapenem-hydrolyzing *β*-lactamases or carbapenemases.

Among these, the *Klebsiella pneumoniae* carbapenemase-2 (KPC-2) belongs to a family of class-A serine (non-metallo) *β*-lactamases, which differs from others by only a few amino acids. The clinical relevance of these enzymes comes from their ability to hydrolyze a broad variety of *β*-lactams, including carbapenems, cephalosporins and penicillins^4^. KPC enzymes have been reported in all Gram-negative members of the “ESKAPE pathogens”^5^ and occur worldwide, although actual prevalence and epidemiological status varies markedly with the country, ranging from sporadic to endemic^6,7^.

These pathogens represent a new paradigm in resistance and pathogenesis and are responsible for life-threatening diseases affecting hospitalized and immune-compromised patients^8,9^. More than 50% of vulnerable patients with hospital-acquired pneumonia die if their infection is caused by a strain producing KPC-2^10,11^. Unfortunately, there is currently no “optimal” treatment to overcome infections caused by KPC-producing bacteria, and clinical outcome data remains sparse^12^. Combinations of antibiotics are most often used, but these must be tailored to match any remaining susceptibility of the infecting strains. New *β*-lactam/*β*-lactamase inhibitor combinations active against KPC (and other) enzymes may offer more generalizable treatment options. One of these combinations, ceftazidime-avibactam, has been licensed by the FDA and EMA^13^. Nevertheless, variant KPC enzymes can be selected or engineered *in vitro* that evade the action even of this new inhibitor combination, although such changes are often associated with decreased carbapenem resistance^14–17^. Moreover, isolates harboring ceftazidime-avibactam-resistant KPC variants have emerged in the clinic^18^. A detailed understanding of the structural characteristics of KPC enzymes may, therefore, allow new inhibitors to be designed.

**Figure 1:**
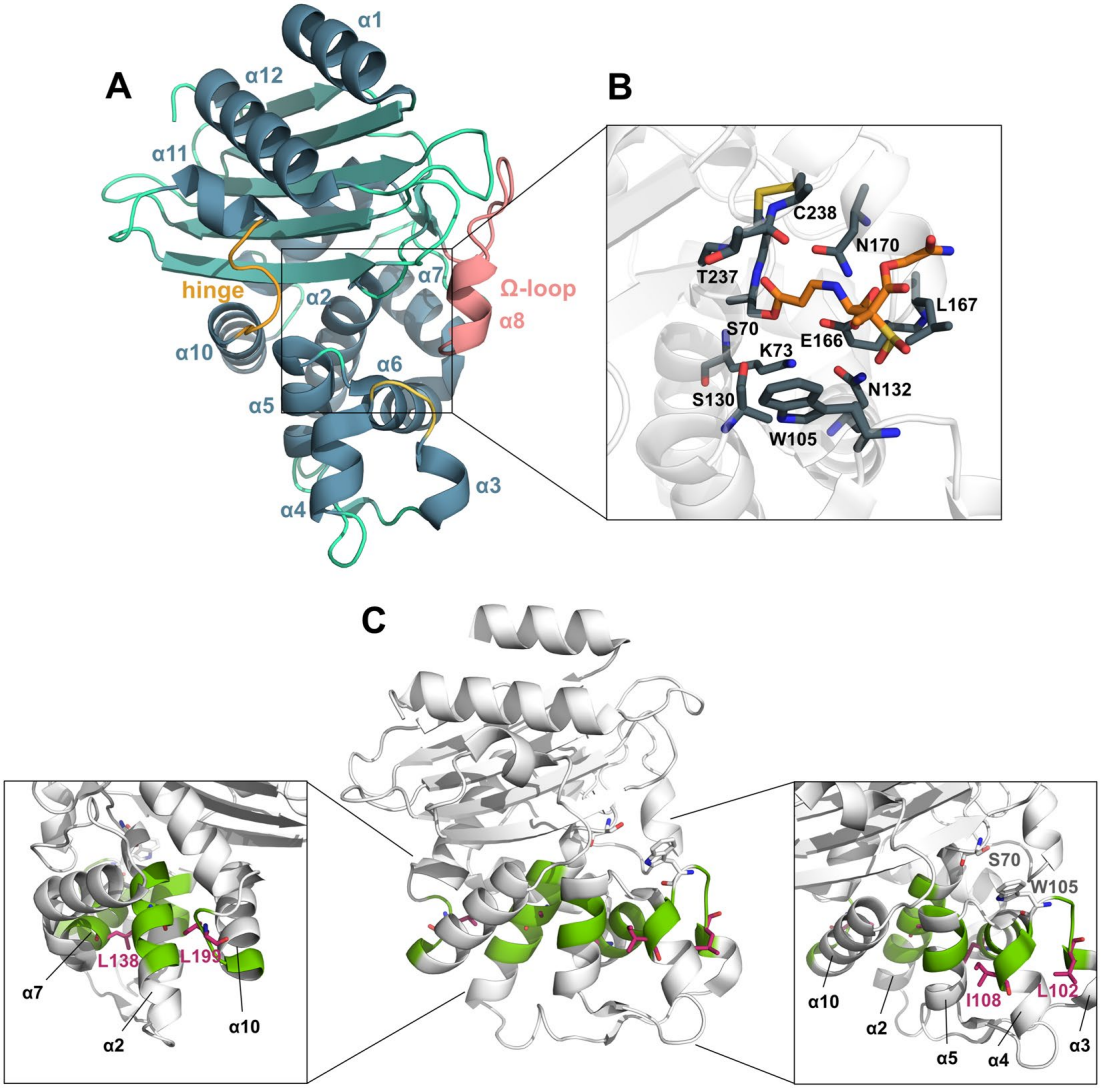
(**A**) Structure of KPC-2 *β*-lactamase. The three loops that surround the active site, i.e. the Ω loop, the hinge region and the loop between *α*3 and *α*4 helices are depicted in pink, orange and yellow respectively. (**B**) Details of the enzyme active site. Residues involved in ligand binding (gray) and the structure of the PSR-3-226 drug (orange), covalently bound to residue S70 have been illustrated as sticks. (**C**) Engineered loss-of-function variants of KPC-2 disrupting the *α*-network. The spatial positions of A77, L102, I108, L138, L199 (red sticks) residues that have been mutated are illustrated. Five single and one double variant have been engineered (A77N, L102T, I108N, L138N, L199R and L102T/I108N). It is rationalized that the hydrophobic interactions between residues of the *α*-network (represented in green) will be disrupted upon mutation.

Structurally, KPC enzymes consist of two sub-domains and the overall fold is similar to that observed in other class-A *β*-lactamases, e.g. SME, SHV and TEM^19^. One sub-domain is mainly *α*-helical, whereas the other contains a five-stranded *β*-sheet, flanked by *α*-helices^20^ (Fig. 1). The cleft, found between the sub-domains, harbors the active site that includes residue S70 and E166 (PDB ID 2OV5), essential for catalysis^21^. It is adjacent to this active site serine that the carbonyl group is positioned and the *β*-lactam ring hydrolyzed^22^. The active site is surrounded by three loops: the Ω loop, which contains residues R164 to D179, the loop between *α*3 and *α*4 helices, where residue W105 is localized and a third loop, also defined as the “hinge region” in TEM-1^23^ that lies opposite to the Ω loop and contains the *α*11 helix turn (Fig. 1). Mutagenesis studies have shown that residue W105, positioned at the perimeter of the active site, plays a crucial role in ligand recognition in KPC enzymes^22,24^ via favorable stacking interactions between the tryptophan side chain and the *β*-lactam ring^25,26^. W105 is not strictly conserved among *β*-lactamases. W105 is replaced by histidine in SME-1 and tyrosine in TEM-1 and SHV-1^27,28^. However, the aromatic nature of this residue is well conserved, supporting the need for a residue that contributes to the stacking and edge to face interactions at the periphery of the active site.

The presence of a disulfide bond (between C69 and C238), and a complex hydrogen bond network ensures the integrity of the enzyme active site^20^. Important clusters of interactions include a pair of conserved lysine residues (K73 and K234) with the N132 and S130 and residue T237, making a hydrogen bond and van der Waals interactions with R220 and H274 respectively. Both R220 and H274, are postulated to affect substrate binding, either electrostatically or indirectly via repositioning of the T237^20^. The ability of KPC to accommodate *β*-substituents of both carbapenems and cephalosporins is thought to be due to subtle but crucial changes in the active site topology, which results in a wider active site compared with TEM-1 and SHV-1^29^.

Understanding the inner regulatory mechanisms, allosteric effects and active site remodeling of KPC enzymes could help to identify critical parameters responsible for its broad-spectrum *β*-lactam resistance and will be crucial to the rational design of effective inhibitors^30–32^. Any delay in the discovery of alternative therapeutic strategies could become critical, as the extended use of inadequate antibiotics has been associated not only with low success rates but with an exacerbation of resistance^33^.

The discovery of covalent inhibitors of KPC-2, such as 3-NPBA (a small boronic acid compound) and PSR-3-226 (a penam sulfone)^34^ and the drug combination ceftazidime-avibactam has been reported as possible strategies to counter the effect of KPC enzymes^13,35^. PSR-3-226 is particularly effective because when bound to KPC-2, it forms a trans-enamine intermediate, which is trapped irreversibly into the active site of the enzyme. Covalent inhibitors have demonstrated a substantial improvement in the stabilization of the binding and result in a 10-fold increased drug affinity with respect to non-covalent inhibitors^34^. Another successful way to tackle KPC-mediated resistance is the use of ceftazidime and avibactam. The addition of avibactam, a non *β*-lactam molecule, to ceftazidime has resulted in a synergistic effect, seen to improve the overall drug efficacy. However, some *in vitro* mutations of KPC found to be resistant to ceftazidime-avibactam, proved how the problem of resistance is still far from being solved^14,15,17,18^.

In this study, we report the identification of “hydrophobic networks” - a system of hydrophobic interactions found to be crucial for structural integrity and allosteric effects of KPC-2, which is one of the most prevalent *β*-lactamase. Long-timescale molecular dynamics simulations, in conjunction with enhanced sampling methods and site-directed mutagenesis experiments provide novel and powerful insights into conformational changes of KPC-2 upon disruption of these networks and ligand binding. Moreover, we emphasize the importance of the novel engineered variants in key regions of KPC-2, that further our understanding of the structural behavior and the regulatory mechanism on which KPC-2 resistance is built. In recent years, a number of KPC-2 variants resulting from single or double amino acid substitutions have been identified in clinical isolates worldwide suggesting a continued evolution of resistance in KPC-2^36,37^. The presence of the newly identified hydrophobic network in these variants and class-A *β*-lactamase family open up new opportunities for the design of small molecules to restore sensitivity to *β*-lactam antibiotics, for which we propose an allosteric approach.

## Results

### The proposed “hydrophobic networks” are highly conserved among class-A *β*-lactamases

**Figure 2:**
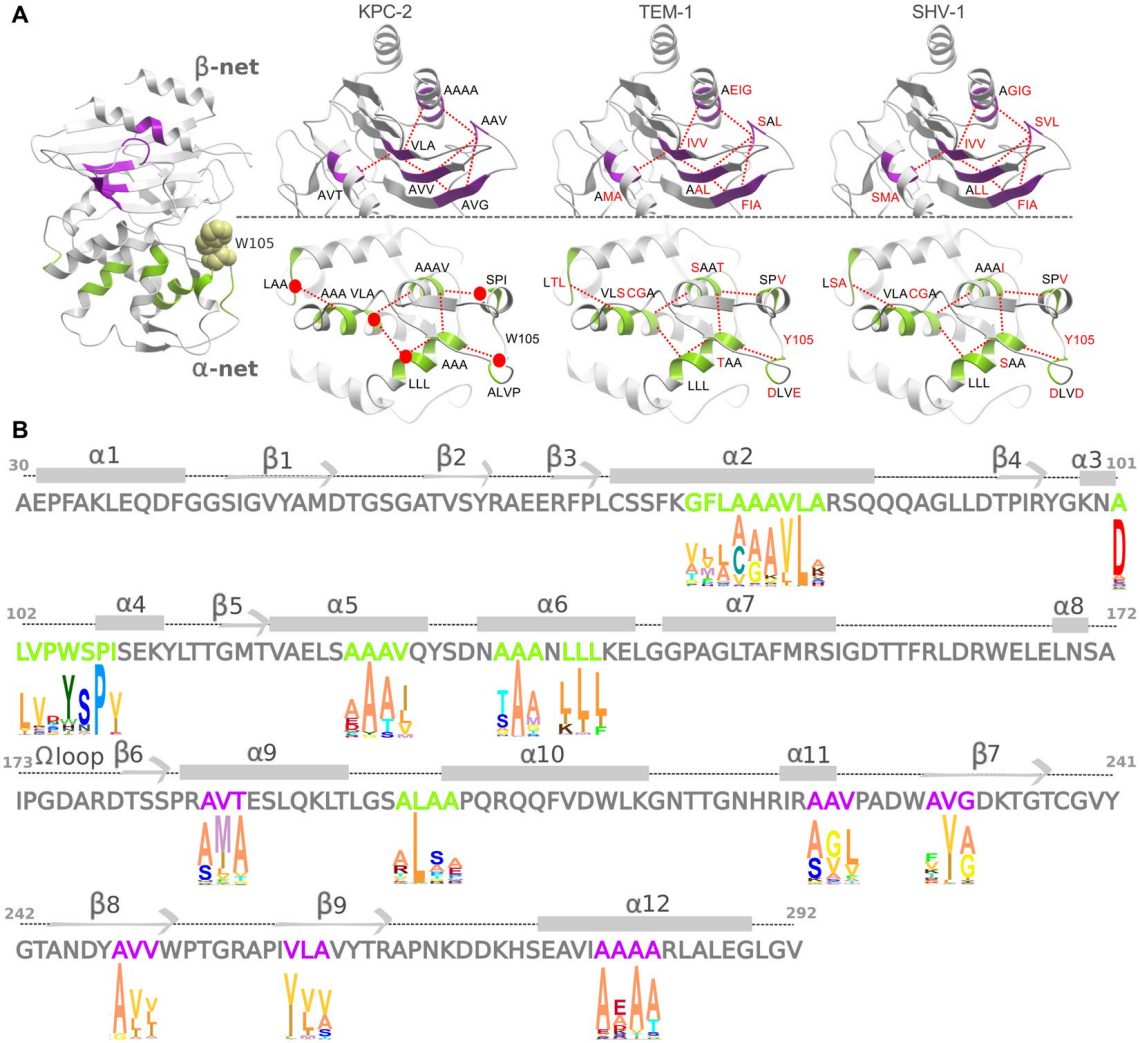
Conservation of “hydrophobic networks” in class-A *β*-lactamases. (**A**) The residues constituting the *β*-network (green) and the *β*-network (lilac) have been highlighted and identified with single letter amino acid code for KPC-2, TEM-1, and SHV-1 structures. Residues not conserved among the three *β*-lactamases have been colored red. The spatial position of the mutated residues (A77N, L102T, I108N, L138N, L199R) has been illustrated as red dots on KPC-2. (**B**) KPC-2 sequence and secondary structure nomenclature used in the text. The numbering has been kept consistent with the 2OV5 PDB structure. The logo plot highlights the conservation of residues in the hydrophobic networks in class-A *β*-lactamase family. The conservation has been derived from an alignment of >80 family members. The height of the letter is proportional to the conservation of the residue. The *α*-network (green) and the *β*-network (lilac) residues have been colored distinctly.

Sequence and structural alignment of KPC-2, TEM-1, and SHV-1 (PDB id: 2OV5, 1XPB and 1SHV respectively) revealed conserved structural elements common to the three proteins. These regions are characterized by repeats of hydrophobic residues (nodes), distributed and connected in a manner that resembles a hydrophobic network (Fig. 2). To investigate whether these “hydrophobic networks” are conserved among class-A *β*-lactamases, we performed a more extensive search based on KPC-2 sequence. The sequence alignment extracted more than 80 non-redundant sequence matches, sharing no less than 26% identity with the target KPC-2 sequence (Supplementary Figs S1, S2, S3).

Despite the high sequence divergence, the alignment shows the identified hydrophobic networks to be strongly conserved among class-A *β*-lactamases. In particular, two hydrophobic networks could be recognized, one in each protein subdomain (Fig. 2). The first network, which we refer to as “*α*-network”, is localized in the helical subdomain and connects the *α*-helices surrounding the active site. The second, which we refer to as “*β*-network”, connects the *β*-sheets of the structure to the C-terminal helix (*α*12) and the loop containing helix *α*11. The actual residues constituting these networks show variation among *β*-lactamases, but their inherent hydrophobic nature remains strongly conserved. Interestingly, a higher sequence variation was observed in the *β*-network than in the *α*-network (Fig. 2). The high conservation rate among class-A *β*-lactamases, highlights the importance of their role, most probably connected with maintaining the structural integrity of the catalytic site, in particular of the *α*-network due to its stronger conservation and its vicinity to the active site. Some of the identified hydrophobic nodes precede or follow other well-known conserved regions in *β*-lactamases that have a direct role in enzyme catalysis (S70-X-X-K73, S130-D-N132) or those that are a part of the active site (K234-T-G236)^38^. The hydrophobic networks seem to ensure the correct positioning of these functional residues, thus having an indirect impact on enzyme function.

Moreover, X-ray structures of TEM-1^39^ and SHV-1^40^ highlighted allosteric inhibitors and crystallographic adjuvants (e.g. Cymal-6) binding to a pocket enclosed between the *α*11 and *α*12 helices. This allosteric pocket is localized exactly in the region corresponding to the identified *β*-network. Given the high hydrophobicity of the pocket, this site remains constricted (cryptic pocket) in the apo enzyme. During the binding, the ligands disrupt the hydrophobic interactions leading to the opening of the allosteric pocket. The “core disruptor” inhibitors of TEM-1, if used alone, are unfortunately not particularly effective (Ki = 480 *μ*M)^39^. However, the researchers have proposed the use of bulkier ligands that could fit better in the allosteric pocket^39^. The connection existing between the active site and the allosteric pocket, studied in TEM-1 in the presence of the BLIP inhibitor, seems to be mostly due to hinge region motions^41^. Between the *α* and *β* networks, we have not identified any direct connection via hydrophobic nodes, thus possibly explaining the weak effect of allosteric inhibitors on the active site. Even if most conventional inhibitors bind to the catalytic site, the existence of core disrupting inhibitors is a proof that allosteric inhibitors targeting pre-formed or cryptic sites affecting the proposed hydrophobic networks might be a viable alternative^30^.

### Disrupting hydrophobic networks restores susceptibility to *β*-lactam antibiotics

What emerges from the sequence alignment and crystal structures analyses is that the identified hydrophobic networks are possibly responsible for the structural stability in class-A *β*-lactamases and the correct positioning of key residues for hydrolysis in the active site (e.g. S70, K73, S130, E166, K234). To test the importance of these networks to resistance, KPC-2 variants containing single and double amino acid substitutions were constructed and examined with minimum inhibitory concentration (MIC) assays for changes in antimicrobial susceptibility. Substituting hydrophobic residues of the identified nodes to hydrophilic ones was hypothesized to disrupt important interactions of the *α*-network. These mutations were chosen based on their predicted importance, being in the vicinity of W105 and forming key contacts within the network. Specifically, the mutated residues L102T and I108N are positioned on helices *α*3 and *α*4, comprising the peripheral loop of the active site, where a recognized hotspot in KPC-2 and other *β*-lactamases, W105, is located (Y105 in TEM-1 and SHV-1). Previous mutagenesis studies have confirmed the importance of W105 in enzyme hydrolysis and structurally coordinating the binding of substrates, not only via stacking and edge-to-face interactions but also by changing its ring orientation adopting a “flipped-in” or “flipped-out” conformations^22,24^. The mutated residue A77N is positioned on helix *α*2 buried in the hydrophobic core of the *α*-helical subdomain of KPC-2, while residue L138N is positioned on helix *α*7. To investigate the effect of perturbations in the *α*-network located far from the catalytic site on the enzymatic activity, we have also mutated L199R, which is positioned on the *α*9–*α*10 loop, approximately 29 Å away from W105. Of the aforementioned, engineered variants, protein of the two single and one double variant has been expressed in *E.coli* DH10B cells: L102T, I108N, and L102T-I108N albeit at a lower expression level than wild-type KPC-2 (Supplementary Fig. S5), while A77N, L138N and L199R were not expressed.

The KPC mediated resistance to carbapenems and cephalosporins for the variants was found to be compromised. Antimicrobial susceptibility testing conducted on *E. coli* strains producing the single variant KPC enzymes (L102T, I108N) and the double variant enzyme (L102T-I108N), showed increased susceptibility to several classes of *β*-lactam antibiotics including penicillins (ampicillin, piperacillin), cephalosporins (ceftazidime, cefotaxime, ceftriaxone, cefepime, cephalothin), monobactams (aztreonam), and carbapenems (imipenem, meropenem, ertapenem, doripenem) and combinations with various classical inhibitors (ampicillin-sulbactam, ampicillin-clavulanate, piperacillin-tazobactam, ceftolozane-tazobactam) compared with wild-type KPC-2 (Table 1). The diazabicyclooctane (DBO) avibactam, an effective KPC inhibitor, when combined with *β*-lactam antibiotics lowered the MICs for both the wild-type strain and variant strains (Table 2), compared with the *β*-lactam alone (Table 1). The loss of function (LOF) observed in the three variants is consistent with the attenuated protein expression, providing evidence for the importance of the *α*-network integrity in protein stability and corresponding resistance. The lack of expression of A77N, L138N and L199R variants could possibly be attributed to misfolding of the enzyme resulting from the lack of tolerance of the polar variant side chains in the hydrophobic microenvironment.

**Table 1:** Susceptibility of *E. coli* strains harboring wild-type pBR322_-cat-blaKPC-2_ and variants to *β*-lactam antibiotics and classical inhibitor combinations.

MIC Values (mg/L)	AMP^a^	PIP^a^	AMP-SUL^a^		AMP-CLA^a^		1 PIP:8 TAZO^a^		AZT^a^	
DH10B	2	2	<0.06		<0.06		4/0.5		0.13	
pBR322 bla_KPC-2_ (WT)	8192	1024	>256		64		1024/128		256	
bla_KPC-2/L102T_	1024	512	16		16		128/16		64	
bla_KPC-2/I108N_	2048	512	16		16		128/16		64	
bla_KPC-2/L102T/I108N_	256	256	4		8		32/8		32	
**MIC Values (mg/L)**	**CAZ** ^**b**^	**TAX** ^**b**^	**CRO** ^**b**^	**FEP** ^**b**^	**THIN** ^**b**^	**TOL-TAZO** ^**b**^	**IMI** ^**b**^	**MER** ^**b**^	**ERT** ^**b**^	**DOR** ^**b**^
**Cephalosporins**							**Carbapenems**			
DH10B	0.3	<0.06	<0.06	<0.125	4	0.25	0.5	<0.06	<0.03	<0.06
pBR322 bla_KPC-2_ (WT)	128	32	128	64	1024	32	8	4	8	4
bla_KPC-2/L102T_	16	4	8	4	512	8	2	0.5	1	1
bla_KPC-2/I108N_	16	4	8	4	512	8	2	0.5	1	1
bla_KPC-2/L102T/I108N_	4	1	1	0.5	512	2	2	0.25	0.30	0.13

^a^Ampicillin (AMP), Piperacillin (PIP), Sulbactam (SUL), Clavulanic acid (CLA), Tazobactam (TAZO), and Aztreonam (AZT).

^b^Ceftazidime (CAZ), Cefotaxime (TAX), Ceftriaxone (CRO), Cefepime (FEP), Cephalothin (THIN), Ceftolozane (TOL), Imipenem (IMI), Meropenem (MER), Ertapenem (ERT), and Doripenem (DOR).

**Table 2:** Susceptibility of *E. coli* strains harboring wild-type pBR322_-cat-blaKPC-2_ and variants to *β*-lactam antibiotics combined with avibactam.

MIC Values (mg/L)	CAZ-AVI^a^	TOL-TAZO-AVI^a^	AZT-AVI^a^	IMI-AVI^a^
DH10B	0.25	0.25	0.13	0.25
pBR322 bla_KPC-2 (WT)_	1	1	0.25	0.5
bla_KPC-2/L102T_	1	0.5	0.25	0.5
bla_KPC-2/I108N_	1	0.5	0.5	0.5
bla_KPC-2/L102T/I108N_	1	0.5	0.5	0.5

^a^Avibactam (AVI), Ceftazidime (CAZ), Ceftolozane (TOL), Tazobactam (TAZO), Aztreonam (AZT), and Imipenem (IMI).

### Engineered variants display altered conformational dynamics

The profound impact the network has on the resistance conferred by the enzyme could be explained only by considering these networks to be structurally involved in the stabilization of overall structure and the catalytic site, thus affecting the regulatory mechanisms of the enzyme which are strictly connected with function. Even if site-directed mutagenesis of the *β*-network has not been explicitly conducted, we could expect a similar behavior as the one obtained for the *α*-network variants, based on the mechanism of action of the allosteric inhibitors of TEM-1. As mentioned previously, the binding of these inhibitors determines the disruption of the hydrophobic core in the *β*-network, able to affect the overall enzyme activity.

To better understand the effect of the introduction of these mutations, we analyzed the changes in the overall dynamics of KPC-2 using atomistic molecular dynamics (MD) simulations of the wild-type (WT) apo structure, the engineered variants and the holo structure with the PSR-3-226 ligand covalently bound to S70, for an aggregate simulation time of 74 *μ*s (Supplementary Table S1). PSR-3-226 was chosen as a model ligand because it is a potent *β*-lactamase inhibitor (Km (KPC-2) = 3.8 *μ*M; Km (SHV-1) = 4.43 *μ*M)^34^ and provides a good start point for further design efforts. The disruption of the communication through the hydrophobic network upon mutation and ligand binding changes the overall flexibility pattern of the WT protein. Root mean square fluctuation (RMSF) analysis performed on the C *α* atoms of KPC-2 of the unbiased simulations showed an increase in the average fluctuations of the regions around the points of mutation and, in all systems, of helix *α*2, helices *α*3-*α*4, and of helix *α*5 (Supplementary Fig. S6). Important residues for catalysis can be found in these regions, such as S70 (*α*2), K73 (*α*2), W105 (*α*3-*α*4), and S130 (*α*5)^21^. Interestingly, the introduction of the A77N mutation led to increased flexibility of areas far from the site of mutation, highlighting the destabilizing nature of this mutation. Analysis of the interactions that each substituted residue is involved with during the course of the unbiased simulations showed that N77 interacts mainly with W210 and S123, rather than with V127, which is instead the main point of interaction of A77 in the WT (Supplementary Fig. S6). The position of A77 on helix *α*2 and in the core of the hydrophobic network (Supplementary Fig. S4), could explain the drastic effect that the mutation in this very position has to the dynamics of the whole enzyme. Moreover, the RMSF analysis showed increased mobility of W105 in the presence of ligand, in agreement with crystallographic data where W105 adopts both “flipped-out” and “flipped-in” conformations and is involved in stacking and edge-to-face interactions with PSR-3-226^34^.

Mutagenesis studies together with computational studies are suggesting that a reduced flexibility of the Ω loop is important for the hydrolytic activity in *β*-lactamases^42,43^. Our analysis indicates that the introduction of the L102T, L102T/I108N, and A77N mutations leads to a higher mobility of residues I172-A176 with respect to the WT. NMR studies performed by Savard *et al*. on the apo form of the highly homologous TEM-1, revealed an overall rigid behavior of the WT structure, with an average S2 of 0.9^44,45^. No relevant motions could be identified by NMR in the order of pico- to nanoseconds timescale, but interestingly slower motions (micro to milliseconds) have been identified for residues of the Ω loop and the active site^42^. Motions on these larger timescales are usually correlated with enzyme catalysis and ligand recognition. To sample slow conformational changes in KPC-2 that might take place on timescales not easily accessible with conventional MD simulations (*μ*s and beyond), and to reconstruct the conformational free energy landscape, multiple-replica parallel tempering metadynamics (PT-metaD) simulations were performed on the WT^46,47^, ligand-bound complex and the variants (Table S1). The free-energy surfaces (FES) for the systems were reconstructed as a function of two collective variables (CVs), chosen based on the analysis performed on the unbiased MD simulations data (Supplementary Fig. S7). CV1 is the distance between W105 and L167 (located in the Ω loop); CV2 is the distance between W105 and T216 (located in the hinge region). These distances aid in sampling the conformational landscape of the three hinge regions surrounding the active site and the movement of the indole ring of residue W105. Two possible conformations of W105 have been proposed based on available X-ray structures^34^: a “flipped-out” conformation, in which the indole group points towards T216 and in which there is a substantial enlargement of the active site cavity, and a “flipped-in” conformation with W105 defining the edge of the pocket and pointing towards L167. The differences in the binding site formed as a result of these conformations could provide invaluable insights into the design of novel inhibitors.

**Figure 3:**
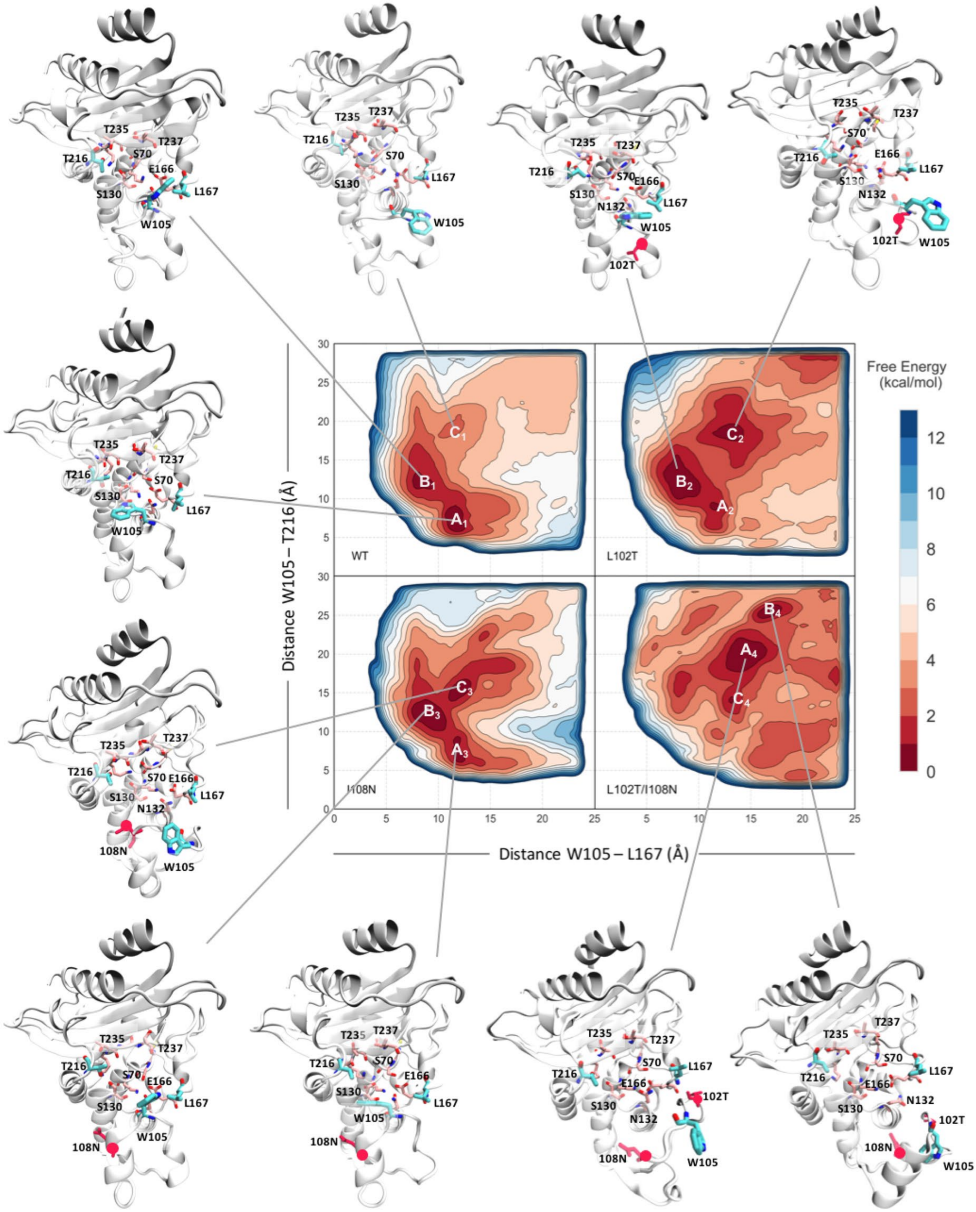
Free-energy surfaces (FES) plots of the WT, KPC-2 single variants L102T, I108N and double variant L102T/I108N. The FES have been derived from the metadynamics simulations and have been reconstructed along the two variables, CV1 (distance between the center of mass of the indole ring of the side chain of W105 and C_*β*_ of L167) and CV2 (distance between the center of mass of the indole ring of the side chain of W105 and C_*β*_ of T216). Structures extracted from their corresponding basins have been illustrated. The mutated residues are shown in red sticks, while residues that have been reported to be important for the catalysis are shown in pink sticks.

The free energy surface (FES) for the WT and the experimentally studied variants of KPC-2 (L102T, I108N, and L102T-I108N) are shown in Fig. 3. In the most stable conformation of the WT, residue W105 adopts the “flipped-out” position, with the indole ring closer in space to T216, while L167 adopts a more solvent exposed position due to a slight opening of the Ω loop (Fig. 3, basin A1). This conformation permits the access to an enlarged active site, ready to accommodate the *β*-lactam, in agreement with mass spectrometry and computational data showing slow timescale motion of the Ω loop and proving its role in hydrolysis^42^. Moreover, WT KPC-2 exhibits an additional local minimum, 2.9 kcal/mol higher than basin A1, where a solvent-exposed conformation of W105 was found (Fig. 3, basin C1). In that conformation, the *α*3-*α*4 hinge region is displaced with respect to the crystal structure and W105 flips away from the active site and points towards the bulk, while the rest of the structure stays intact.

In the variants L102T, I108N, and L102T-I108N the conformational landscape of KPC-2 changes due to the perturbation of the *α*-network. The introduction of the T102 and N108 single mutations seem to tip the balance of the conformational equilibrium towards conformations where the *α*3-*α*4 hinge region has flipped towards the bulk. Specifically, in the most stable conformation of the L102T (Fig. 3, basin C2), the sidechain of T102 forms a hydrogen bond with the backbone of G98, while the interaction of N132 with the backbone of P104 and W105, which seems to keep the *α*3-*α*4 hinge region in place in the WT, is now not lost in the variant. Similarly to L102T, the most stable conformation of I108N corresponds to the one where the *α*3-*α*4 hinge region is deformed, and W105 does not define the edge of the binding site anymore (Fig. 3, basin C3). In this conformation, W105 is stacked with P107, while the N108 and Y102 side chains are closely juxtaposed. Even though the crystallographic poses are still accessible by both L102T and I108N variants, with W105 adopting the flipped-in (Fig. 3, basins B2, B3) and flipped-out conformations (Fig. 3, basins A2, A3), the stabilization of conformations where the *α*3-*α*4 hinge region is partially detached and W105 flips towards the bulk could explain the loss of enzyme resistance inferred by the MIC experiments reported previously (Table 1). In the case of the double variant L102T-I108N, the stabilized FES minima differ even more from those of the WT. The enzyme is now unable to explore the crystallographic poses, primarily because of major structural rearrangements caused by the disruption of the *α*-network by mutations. In the WT structure, helices *α*3 and *α*4 remain in contact with helices *α*5 and *α*6 by maintaining strong interactions between key residues of SPI-AAAV and ALVP-AAA, identified as nodes of the network (Fig. 2). In this conformation, helices *α*3 and *α*4 can shape the active site of the enzyme and can control the entrance of ligands through the displacement of residue W105. However, in the most stable conformation of the double variant (Fig. 3, basin A4), helices *α*3 and *α*4 have unfolded, and they have been partially detached from helices *α*5 and *α*6. The newly visited conformations are clearly not stable in the WT enzyme.

To further investigate the effect of *α*-network perturbations on the structure of the KPC-2, additional variants spread throughout the nodes of the *α*-network were studied *in silico*. In our mutagenesis experiments, A77N, L138N and L199R were not expressed (Supplementary Fig. S5). It is highly likely that these mutations lead to loss of function by destabilizing the entire structure as they are spatially positioned in a highly hydrophobic environment. Surprisingly, the introduction of the N77 mutation did not seem to change the free energy landscape of the variant enzyme in the phase space of the two selected CVs (Supplementary Fig. S8). However, the increased flexibility that was found from the unbiased simulations is reflected in the broadening of the visited basins. On the other hand, variant L138N, although it is still able to access conformations very similar to the WT, it also stabilizes an inactive conformation (Supplementary Fig. S8, basin D6) in which the *α*3-*α*4 region loses contact with the rest of the enzyme.

Interestingly, the L199R mutation seems to initiate a profound destabilization on the arrangement of the active site, despite the fact that L199R is located the furthest away from the catalytic site of all variants (29Ã). Specifically, when L199, which is located at N-terminal of helix *α*10, is mutated, the hinge region, which is located in the C-terminal of helix *α*10, flips away from the catalytic site and points towards the bulk. Moreover, the FES of the L199R variant exhibits another local minimum (Supplementary Fig. S8, basin D7), where the *α*3-*α*4 region has shifted laterally towards L167 and is entirely detached and exposed to the solvent. The variant shows a smooth diffusion among the different conformations, with almost no energy barriers between them (less than 1 kcal/mol). The effect of L199R to the integrity of the catalytic site, given its position, indicates that the identified hydrophobic networks is a promising target for drug discovery and gives hope for successful attempts of allosteric inhibitors design.

Contrary to our original assumption that disruption of the *α*-network would occur as a consequence of irreversible binding by PSR-3-226, the structural analysis of the conformations the holo structure mainly visits showed that the “hydrophobic networks” are present and stable. In the most stable conformation (Supplementary Fig. S8, basin B8), PSR-3-226 forms a hydrogen bond with N136 via its carboxyl group and hydrophobic interactions with L167 via its methyl group, while the acetamido group of PSR-3-226 is stacked on top of W105, which adopts the “flipped-in” conformation. It is, therefore, reasonable to believe that PSR-3-226 exerts its inhibitory activity through its irreversible binding to the active site that deters antibiotics from binding, rather than through a structural disruption.

### Disruption of *α*-hydrophobic network impacts the conformation of the active site

Docking of the experimentally studied antibiotic cefepime to the most stable conformation of the WT further supports the important role of W105 in the stabilization of the substrate in the catalytic site. In this conformation, W105 adopts the “flipped-out” orientation and it was found to provide favorable stacking interactions with the *β*-lactam ring of cefepime, which in turn led to an orientation of the *β*-lactam carbonyl group towards S70 in a conformation that resembles the reported tetrahedral intermediate of the first acylation step of the *β*-lactam ring hydrolysis (Supplementary Fig. S9)^21^. On the contrary, docking of cefepime to the “flipped-in” conformation, where the side chain of W105 is proximal to L167 (Fig. 3, basin B1), showed that the cephalosporin substrate is mainly stabilized through interactions of its carboxyl moiety with the side chain of T237, and through two hydrogen bonds between the amide and carbonyl group of cefepime and the backbone amide group of T237, whose amide group is involved in the oxyanion hole together with the backbone nitrogen of S70 (Fig. S9). It has been reported that the interaction between the backbone amide hydrogens of the oxyanion hole and the carbonyl oxygen of the antibiotics becomes increasingly stabilizing as the nucleophilic attack proceeds 21. Although it is not possible to obtain qualitative results concerning the hydrolysis mechanism of KPC-2 via our computational approach, the presence of the two minima in the FES of the WT and the corresponding binding poses of cefepime to these minima indicate that W105 may change conformation to further stabilize different intermediates of the catalytic reaction.

All the docking poses of cefepime to the most stable conformation of L102T, in which the *α*3-*α*4 hinge region has flipped towards the bulk (Fig. 3, basin C2), resulted in much lower docking scores compared to the ones in the WT (21 compared to 14). In the highest scored pose, the carboxyl group of the substrate forms a hydrogen bond with the side chain of S70, as well as two hydrogen bonds between its lactam oxygen and amide group and the backbone of T237 (Supplementary Fig. S9). However, the antibiotic is much more solvent exposed compared to the WT pose, and it is highly likely that cefepime binds to the catalytic site only transiently, forming unfavorable interactions for the catalysis, which explains the almost complete inability of the mutated enzyme to hydrolyze cefepime (Table 1). In the case of the L102T/I108N variant, where helices *α*3 and *α*4 have unfolded, hydrogen bonds between the carbonyl group of the *β*-lactam ring of cefepime and the amide group and backbones of S70 and T237 are formed, as well as two hydrogen bonds between the side chain of T237 and the carboxyl and amide groups of cefepime (Fig. S9). Same as in the case of L102T, the deformation of helices *α*3 and *α*4 leaves cefepime solvent exposed.

Based on our observations from both simulations and experiments, we rationalize that the drastic loss of KPC-2 function and restored *β*-lactam sensitivity is due to active site disruption, resulting from a loss of structural integrity and allosteric signaling, subsequent to “hydrophobic network” breakage upon mutation.

## Discussion

Class-A *β*-lactamases exhibit a conserved structure that has evolved into a highly efficient hydrolytic engine for the rapid inactivation of *β*-lactam antibiotics. The original results presented herein, identify conserved structural elements reported for the first time, which we refer to as “hydrophobic networks”, found to be strongly conserved among class-A *β*-lactamases and with the potential to provide a much-needed rationale for the design of allosteric drugs against bacterial resistance.

The reported high degree of conservation among the class-A family indicates these residues to be somehow essential for the enzyme and to have been selected over evolution. As proven by both simulations and experiments, the hydrophobic networks assure structural integrity and they guarantee the right positioning of residues in the active site essential for catalysis (e.g. S70-X-X-K73), thus being indirectly correlated to enzyme-mediated resistance. When variants of KPC-2 that disrupt the *α*-network were tested, resistance to carbapenems and cephalosporines was shown to be compromised. The role played by hydrophobic interactions in protein folding, tertiary structure stability, and allostery is well established^48–51^, as well as the existence of functional hydrophobic networks in proteins. One example is the “hydrophobic spines” identified in protein kinases, another biological target of primary importance^52–54^. The spines of kinases are a perfect example of hydrophobic connections having a profound impact on protein architecture and function.

KPC-2 along with other class-A *β*-lactamases were found to present two spatially conserved hydrophobic networks, the *α*-network, and the *β*-network, embedded into the *β*-lactamase structure. We propose these “networks” to be critical constituents of class-A *β*-lactamases architecture and through that to be associated with resistance. The natural stability and persistence of these networks over time in WT KPC-2 have been demonstrated in our simulations, but it is also supported by NMR experiments performed on highly homologous TEM-1, identifying it as one of the most ordered and stable protein^55,56^. The underlying architecture of the domains, provided by the integrity of these networks is a requisite that if not satisfied, as in the case of the engineered variants in the study, leads to loss of enzyme functionality, and so to a loss of resistance.

Both simulations and experiments carried out on the designed LOF variants have highlighted the link between KPC-2 *β*-lactam susceptibility and the hydrophobic networks integrity. It is clear that disruption of the hydrophobic network as well as their modulation through ligand-binding events result in a profound alteration in the dynamics of KPC-2, probably associated with a certain loss in structural integrity. From our detailed investigation of the conformational changes of W105, spatially positioned at the periphery of the enzyme active site, we can also postulate that the conformation in which W105 has flipped out, is more stable and so is the one which is most likely to be encountered by a ligand during engagement. On the contrary, conformations that are stabilized upon mutation in which W105 points towards the bulk could explain enzyme’s loss of function as helices *α*3 and *α*4 can no longer shape the active site of the enzyme and therefore can not control the entrance of ligands through the displacement of residue W105. Furthermore, the enlargement of the active site seen in WT KPC-2 when no ligand is bound and determined by the concerted motion of the loop where W105 is located and the Ω loop, suggests the possible accommodation of larger ligands in the active site.

Moreover, as in the case of the core disruptor inhibitors of TEM-1, a feasible way to inhibit class-A *β*-lactamases is by disrupting the folded structure of the enzyme. Probably ligands, which affect structural instability, could also be a route to inhibition. With this perspective, based on the collected data in this study and in particular on the recovered *β*-lactam sensitivity in the engineered *α*-network disruptor variants, we propose the hydrophobic networks as a possible innovative way to target not only KPC variants (Supplementary Fig. S10), but all class-A *β*-lactamases in general, in a selective and effective manner.

The insights on structure and regulatory mechanisms of class-A *β*-lactamases elucidated in this work could determine advances in the field and have a significant impact on the development of future effective therapies against bacterial resistance.

## Methods

### Sequence alignment and evolutionary tree generation

The sequences were selected using the HMMER web server (website version 1.8)^57^. The sequence search was performed with the SwissProt database^58^. The 83 sequence matches were obtained in the first iteration, 86 in the second iteration. After checking the divergence (not less than 26% of sequence identity), the obtained sequences were aligned and analyzed with the Jalview program version 2^59^. The alignment has been tailored eliminating redundancy and not significant sequences (two eliminated), then colored using the ClustalX option (Supplementary Figs S1, S2, S3). The logo plot has been generated using the Skylign website (skylign.org). The phylogenetic tree was created by iTOL (http://itol.embl.de) using default parameters.

### Computational methods

The KPC-2 structures of the wild-type and covalently-bound ligand were retrieved from the Protein Data Bank (PDB id 2OV5 and 3RXW respectively^20,34^). The variants were constructed *in silico* using the ICM mutagenesis program^60^. Amber ff14SB force field^61,62^ was used to describe protein interactions in the system, with explicit TIP3P water molecules^63^. The protonation state of the residues was determined both using PROPKA 3.1 and PDB2PQR 2.1 as implemented in the proteinPrepare() functionality of HTMD^64^. The charges of PSR-3-226 were calculated at the DFT level of theory using B3LYP hybrid functional and 6–31 G* basis set with the Gaussian03 package and the parameters for the rest of the bonded and non-bonded interactions of the ligand were adopted from the GAFF2 force field^65^. All systems were minimized with 1000 steps of steepest descent integrator and equilibrated in the NPT ensemble for 1 ns, using a Berendsen barostat at 1 atm^66^ with initial velocities sampled from the Boltzmann distribution at 300 K. The temperature was kept at 300 K by a Langevin thermostat. The PME algorithm was used for electrostatic interactions with a cut-off of 1.2 nm. A 1 *μ*s production run was carried out for all the systems in the NVT ensemble with hydrogen mass repartitioning that allowed for a time step of 4 fs. To enhance the conformational sampling, additional adaptive simulations that favor the exploration of new states were performed as implemented in the ACEMD program^67,68^. Each adaptive run consisted of e = 10 epochs, each consisting of N = 4 parallel simulations of 300 ns and the simulation parameters were kept consistent with the previous runs. During the adaptive simulations, a rough Markov model^69^ is built on the fly and used to decide from which part of the conformational space to spawn the next simulations. In the adaptive simulations, we used the distance between the C*α* atoms of the residues that are involved in the hydrophobic nodes as a feature, the slowest linear combinations were computed by the tICA method (lag time 200 ns)^70^, and this space was discretized using k-means clustering (k = 200). All four runs per epoch use the same conformation derived from the Markov model for their first epoch, but after that they diverge since they run independently. To study the W105 transition, we chose a set of CVs consisted of the following two distances: CV1 = distance (<W105>, L167(C_*β*_)) and CV2 = distance (<W105>, T216(C_*β*_)), where <> denotes the center of mass of the indole group (Supplementary Fig. S7). The distributions calculated from unbiased simulations are illustrated in Supplementary Fig. S11.

To sample conformations separated by high energy barriers, which may not had been visited using unbiased simulations, we performed parallel tempering well-tempered metadynamics^71,72^ (PT-WTmetaD) with eight replicas per system at increasing temperatures (300, 302, 305, 307, 310, 312, 315 and 317 K) of the KPC-2 WT, ligand-bound and variant structures using Desmond 3.6^73^. A total of 2.4 *μ*s in the NVT ensemble were needed to reach full convergence of the free energy. The sampling convergence was checked by comparing the reconstructed free energy surfaces at different time intervals during the last 50 ns of the simulations. Production simulations were initiated from the final snapshot of the corresponding equilibration simulations. All bond lengths of hydrogen atoms were constrained using M-SHAKE^74^. An r-RESPA integrator was used with a time step of 2 fs for the short-range bonded and non-bonded interactions, and long-range non-bonded interactions were computed every 6 fs^75^. The bias factor was determined by the kT value in the corresponding sampling temperature and the initial Gaussian height to 0.3 kcal/mol, deposited every 1 ps. The Gaussian width was set to 0.2 and 0.2 Å for the two CVs, respectively. The free energy surface of the metadynamics simulation as a function of the two CVs is readily obtained by integrating the deposited energy bias along the trajectory. The structures corresponding to the minima were selected from the metadynamics trajectories, based on the values of collective variables CV1 and CV2. To retrieve representative structures of the minima, the conformations corresponding to each minimum on the 300 K replica were clustered based on C *α* RMSD. The representative conformations of the most highly populated clusters are depicted in Fig. 3 and Supplementary Fig. S9.

The antibiotic cefepime (a fourth-generation cephalosporin), which was experimentally used to measure the hydrolytic activity of KPC-2 WT and the variants, was docked to the representative structures of the basins that were identified through the metadynamics simulations using the ICM software^60^. Flexible ligand docking with the ICM software uses Monte Carlo simulations to globally optimize a set of internal ligand coordinates in the space of grid potential maps calculated for the protein catalytic site. The automatically determined Monte Carlo run length was extended by a multiplier (thoroughness) of five, and five docking conformations were obtained. The unbiased simulations were analyzed using functionalities available on PyEMMA^76^ and MolPX packages. The RMSF (Supplementary Fig. S4) was calculated from the whole set of unbiased simulations of the WT, variants and ligand-bound structures, using the gmx_rmsf tool of the GROMACS 5.1.2 package^77^. The first 150 ns of each simulation were considered equilibration time and omitted from the calculation. The structural figures were generated using VMD, ICM-Pro and PyMol software^60,78^.

### Experimental methods

#### Site-saturation Mutagenesis

*E. coli* containing bla_KPC-2_ in pBR322-catI vector was a gift from Dr. Fred Tenover previously of the Centres for Disease Control and Prevention (Atlanta, GA)^79^. Mutagenesis was performed at selected nucleotide positions in bla_KPC-2_ in the pBR322-catI plasmid using degenerate primers and the Quikchange Site-Directed Mutagenesis Kit (Agilent Technologies) per the manufacturer’s instructions. Resulting plasmids were transformed into *E. coli* DH10B Electromax cells (Invitrogen). All nucleotide sequences in the bla_KPC-2_ gene were confirmed by sequencing (Molecular Cloning Laboratories, McLab, South San Francisco, CA) using bla_KPC-2_ primers.

#### Protein Expression of wild-type KPC-2 and variants

Immunoblotting was performed to assess protein expression in whole cell 22 and periplasmic extracts 80 as previously described (Supplementary Fig. S5). *E. coli* cells were grown to an OD600 = 0.7 to 0.8 using chloramphenicol to maintain the plasmid. One OD600 U of cells was pelleted at 10,000 rpm for 5 min. The supernatant was removed, and the pellet was frozen at −20 °C. To prepare whole cell lysates, each pellet was resuspended directly in 50 *μ*l of 5x sodium dodecyl sulfate-polyacrylamide gel electrophoresis (SDS-PAGE) loading dye and boiled for 10 minutes. Samples were vortexed rigorously. A volume of 10 *μ*l of cell suspension was loaded onto each lane of a 10% SDS gel. For periplasmic extracts, each cell pellet was lysed in 100 *μ*l 50 mM Tris-HCl, pH 7.4, with lysozyme, benzonuclease, MgSO_4_, and EDTA on a shaking platform, mixed with 5x SDS-PAGE loading dye, vortexed, and boiled for 10 minutes. Equal volumes of periplasmic extract were loaded onto each lane of a 10% SDS-PAGE gel.

The proteins were separated on the SDS-PAGE gel and transferred to polyvinylidene fluoride (PVDF) membrane. Non-specific binding sites on the membrane were blocked at least one hour with 5% milk in 25 mM Tris-buffered saline (TBS) pH 7.4. The membrane was probed using an *α*-KPC-2 antibody (1:5,000) and *α*-DnaK (1:30,000, *E. coli* mAb, Enzo Life Science) as a protein loading control in 5% milk in TBS for 3 hours at room temperature (or overnight at 4 °C). After at least ten washes in TBS with 0.05% Tween 20 (TBS-T) for 10 minutes each, secondary antibodies (protein goat HRP, Bio-Rad and goat anti-mouse IgG-HRP, Santa Cruz) were used at 1:10,000 and 1:30,000 respectively in 5% milk in TBS for 1 hour at room temperature. The Western blot was rewashed in TBS-T at least ten times for 10 minutes each and developed with the Amersham Prime ECL Reagent Kit (GE Healthcare) and Fotodyne Luminary/FX workstation imaging system.

#### Antimicrobial susceptibility

Mueller-Hinton (MH) agar-dilution minimum inhibitory concentration (MIC) measurements were performed according to Clinical and Laboratory Standards Institute (CLSI) guidelines 2014, Performance Standards for Antimicrobial Susceptibility Testing; Twenty-Fourth Informational Supplement^80,81^. The MICs are reported as the concentrations at which bacterial growth was no longer observed. Avibactam was tested at a constant 4 *μ*g/mL in combination with its respective antibiotic partners. All MIC measurements were performed at least three times.

Ampicillin, piperacillin, ceftriaxone, cephalothin, potassium clavulanate, cefotaxime, and chloramphenicol were purchased from Sigma-Aldrich. Ceftazidime was procured from Sigma and Research Products International and used interchangeably throughout the experimentation. Imipenem was obtained from USP and from the commercial source. Sulbactam was bought from Astatech. Tazobactam and aztreonam were purchased from Chem-Impex Int’l. Ceftolozane-tazobactam, cefepime, meropenem, ertapenem, and doripenem were obtained from their commercial sources. Avibactam was purchased from Advanced ChemBlocks.

## Electronic supplementary material

Supplementary Information
