# Supplementary material for: Defining the architecture of KPC-2 Carbapenemase: identifying allosteric networks to fight antibiotics resistance
Source: Sci Rep. 2018 Aug 27;8:12916. doi: 10.1038/s41598-018-31176-0 (PMC6110804; doi:10.1038/s41598-018-31176-0)
Supplement: Supplementary file 1 — Supplementary Information [file 41598_2018_31176_MOESM1_ESM.pdf]

SUPPLEMENTARY INFORMATION

Defining the architecture of KPC-2 carbapenemase: identifying allosteric networks to fight antibiotic resistance

Ioannis Galdadas[1#], Silvia Lovera[1#], Guillermo Pérez-Hernández[3], Melissa D Barnes[4], Jess Healy[5], Hamidreza Afsharikho[5], Neil Woodford[6], Robert A Bonomo[4], Francesco L Gervasio[1,2], Shozeb Haider[5*]

[1]Department of Chemistry, University College London, London, UK
[2]Institute of Structural and Molecular Biology, University College London, London, UK
[3]Department of Mathematics and Computer Science, Freie Universitat Berlin, Berlin, Germany
[4]Department of Medicine, Case Western Reserve University & Research Service, Louis Stokes Cleveland Department of Veterans Affairs,, Cleveland, Ohio
[5]UCL School of Pharmacy, University College London, London, UK
[6]Public Health England, London, UK

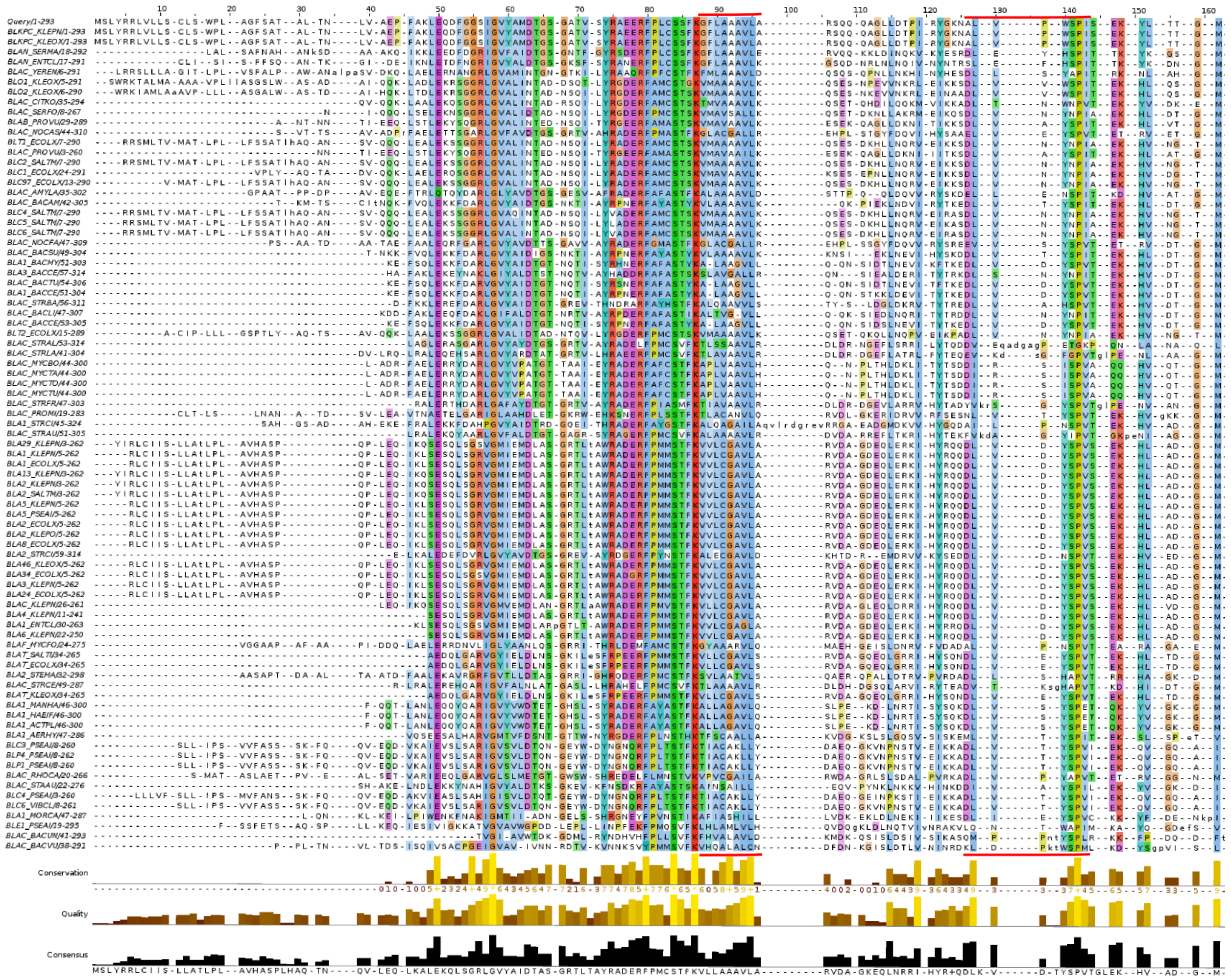

Supplementary Figure S1: Sequence alignment of 83 sequence matches obtained with the Jalview program (version 2) using KPC-2 sequence (2OV5.pdb) as query. The conserved residues are highlighted and the colouring is based on their chemical nature. Red bars have been included to highlight the position of residues in the hydrophobic networks.

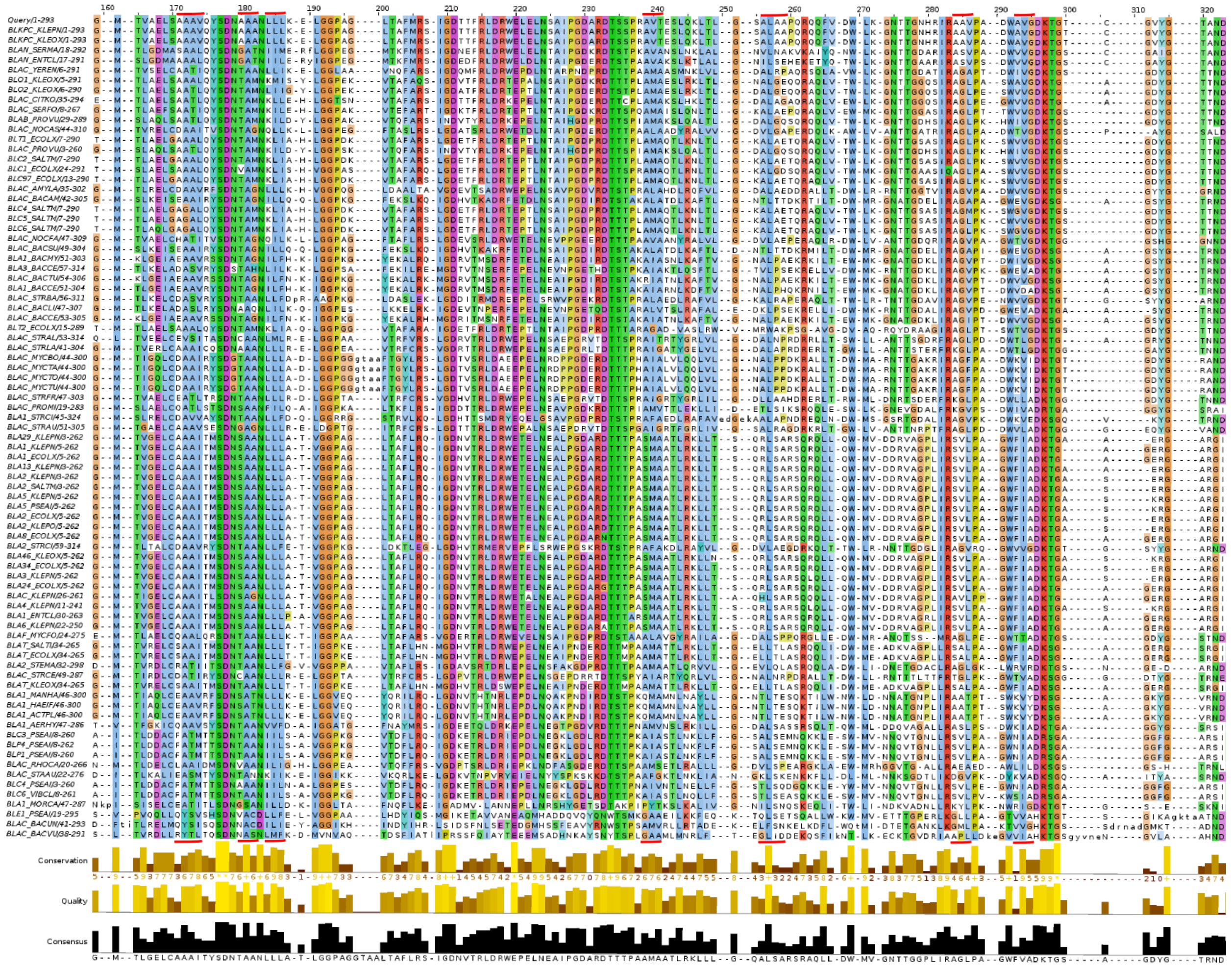

Supplementary Figure S1 (continued): Sequence alignment of 83 sequence matches obtained with the Jalview program (version 2) using KPC-2 sequence (2OV5.pdb) as query. The conserved residues are highlighted and the colouring is based on their chemical nature. Red bars have been included to highlight the position of residues in the hydrophobic networks.

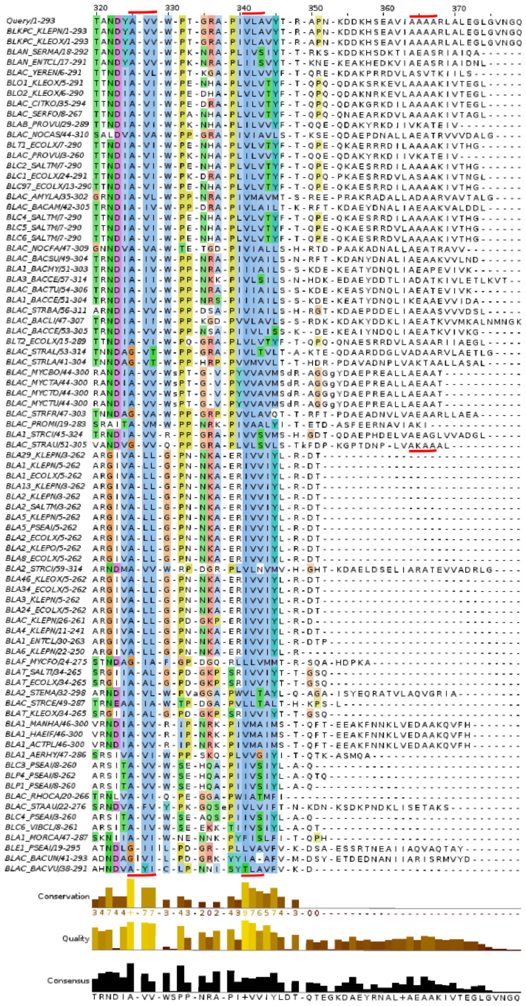

Supplementary Figure S1 (continued): Sequence alignment of 83 sequence matches obtained with the Jalview program (version 2) using KPC-2 sequence (2OV5.pdb) as query. The conserved residues are highlighted and the colouring is based on their chemical nature. Red bars have been included to highlight the position of residues in the hydrophobic networks.

Target	Description	Species	E-value
BLKPC_KLEPN	Carbapenem-hydrolyzing beta-lactamase KPC	Klebsiella pneumoniae	1.2e-193
BLKPC_KLEOX	Carbapenem-hydrolyzing beta-lactamase KPC	Klebsiella oxytoca	1.2e-193
BLAN_SERMA	Carbapenem-hydrolyzing beta-lactamase Sme-1	Serratia marcescens	6.9e-104
BLAN_ENTCL	Imipenem-hydrolyzing beta-lactamase	Enterobacter cloacae	3.9e-100
BLAC_YEREN	Beta-lactamase	Yersinia enterocolitica	9.3e-86
BLO1_KLEOX	Beta-lactamase OXY-1	Klebsiella oxytoca	1.3e-85
BLO2_KLEOX	Beta-lactamase OXY-2	Klebsiella oxytoca	1.3e-84
BLAC_CITKO	Beta-lactamase	Citrobacter koseri	5.6e-84
BLAC_SERFO	Beta-lactamase	Serratia fonticola	3.5e-82
BLAB_PROVU	Beta-lactamase	Proteus vulgaris	7.2e-81
BLAC_NOCAS	Beta-lactamase AST-1	Nocardia asteroides	4.6e-80
BLT1_ECOLX	Beta-lactamase Toho-1	Escherichia coli	1.6e-79
BLAC_PROVU	Beta-lactamase	Proteus vulgaris	1.6e-79
BLC2_SALTM	Beta-lactamase CTX-M-2	Salmonella typhimurium	2.2e-79
BLC1_ECOLX	Beta-lactamase CTX-M-1	Escherichia coli	4.4e-79
BLC97_ECOLX	Beta-lactamase CTX-M-97	Escherichia coli	5.5e-79
BLAC_AMYLA	Beta-lactamase	Amycolatopsis lactamdurans	1.8e-78
BLAC_BACAM	Beta-lactamase	Bacillus amyloliquefaciens	6.1e-78
BLC4_SALTM	Beta-lactamase CTX-M-4	Salmonella typhimurium	3.0e-77
BLC5_SALTM	Beta-lactamase CTX-M-5	Salmonella typhimurium	3.8e-77
BLC6_SALTM	Beta-lactamase CTX-M-6	Salmonella typhimurium	6.3e-77
BLAC_NOCFA	Beta-lactamase FAR-1	Nocardia farcinica (strain IFM 10152)	1.7e-75
BLAC_BACSU	Beta-lactamase	Bacillus subtilis (strain 168)	5.1e-72
BLA1_BACMY	Beta-lactamase 1	Bacillus mycoides	3.1e-70
BLA3_BACCE	Beta-lactamase 3	Bacillus cereus	1.0e-69
BLAC_BACTU	Beta-lactamase	Bacillus thuringiensis	1.2e-69
BLA1_BACCE	Beta-lactamase 1	Bacillus cereus	1.9e-69
BLAC_STRBA	Beta-lactamase	Streptomyces badius	1.7e-68
BLAC_BACLI	Beta-lactamase	Bacillus licheniformis	2.0e-67
BLAC_BACCE	Beta-lactamase 1	Bacillus cereus	2.5e-67
BLT2_ECOLX	Beta-lactamase Toho-2	Escherichia coli	3.3e-67
BLAC_STRAL	Beta-lactamase	Streptomyces albus G	7.1e-67
BLAC_STRLA	Beta-lactamase	Streptomyces lavendulae	5.2e-66
BLAC_MYCBO	Beta-lactamase	Mycobacterium bovis	1.7e-63
BLAC_MYCTA	Beta-lactamase	Mycobacterium tuberculosis (strain ATCC 25618 / H37Rv)	1.7e-63
BLAC_STRFR	Beta-lactamase	Streptomyces fradiae	1.9e-63
BLAC_PROMI	Beta-lactamase	Proteus mirabilis	6.4e-63
BLA1_STRCI	Beta-lactamase 1	Streptomyces cacaoi	2.3e-62
BLAC_STRAU	Beta-lactamase	Streptomyces aureofaciens	6.1e-62
BLA29_KLEPN	Beta-lactamase SHV-29	Klebsiella pneumoniae	8.3e-62
BLA1_KLEPN	Beta-lactamase SHV-1	Klebsiella pneumoniae	2.8e-61
BLA1_ECOLX	Beta-lactamase SHV-1	Escherichia coli	2.8e-61
BLA13_KLEPN	Beta-lactamase SHV-13	Klebsiella pneumoniae	4.0e-61
BLA2_KLEPN	Beta-lactamase SHV-2	Klebsiella pneumoniae	4.8e-61
BLA2_SALTM	Beta-lactamase SHV-2	Salmonella typhimurium	4.8e-61
BLA5_KLEPN	Beta-lactamase SHV-5	Klebsiella pneumoniae	9.2e-61
BLA5_PSEAI	Beta-lactamase SHV-5	Pseudomonas aeruginosa	9.2e-61
BLA2_ECOLX	Beta-lactamase SHV-2	Escherichia coli	9.2e-61
BLA2_KLEPO	Beta-lactamase SHV-2	Klebsiella pneumoniae subsp. ozaenae	9.2e-61
BLA8_ECOLX	Beta-lactamase SHV-8	Escherichia coli	1.4e-60
BLA2_STRCI	Beta-lactamase 2	Streptomyces cacaoi	1.5e-60
BLA46_KLEOX	Beta-lactamase SHV-46	Klebsiella oxytoca	1.7e-60
BLA34_ECOLX	Beta-lactamase SHV-34	Escherichia coli	1.8e-60
BLA3_KLEPN	Beta-lactamase SHV-3	Klebsiella pneumoniae	2.5e-60
BLA24_ECOLX	Beta-lactamase SHV-24	Escherichia coli	2.7e-60
BLAC_KLEPN	Beta-lactamase	Klebsiella pneumoniae	5.2e-60
BLA4_KLEPN	Beta-lactamase SHV-4	Klebsiella pneumoniae	7.1e-60
BLA1_ENTCL	Beta-lactamase Ohio-1	Enterobacter cloacae	1.5e-59
BLA6_KLEPN	Beta-lactamase SHV-6 (Fragment)	Klebsiella pneumoniae	1.9e-59
BLAF_MYCFO	Beta-lactamase	Mycobacterium fortuitum	2.0e-58
BLAT_SALTI	Beta-lactamase TEM	Salmonella typhi	3.4e-58
BLAT_ECOLX	Beta-lactamase TEM	Escherichia coli O111:H- (strain 11128 / EHEC)	3.4e-58
BLA2_STEMA	Beta-lactamase L2	Stenotrophomonas maltophilia	3.8e-58
BLAC_STRCE	Beta-lactamase	Streptomyces cellulosae	4.4e-58
BLAT_KLEOX	Beta-lactamase TEM-12	Klebsiella oxytoca	2.4e-57
BLA1_MANHA	Beta-lactamase ROB-1	Mannheimia haemolytica	5.9e-57
BLA1_HAEIF	Beta-lactamase ROB-1	Haemophilus influenzae	5.9e-57
BLA1_ACTPL	Beta-lactamase ROB-1	Actinobacillus pleuropneumoniae serotype 7 (strain AP76)	5.9e-57
BLA1_AERHY	Beta-lactamase AER-1	Aeromonas hydrophila	3.1e-55
BLC3_PSEAI	Beta-lactamase CARB-3	Pseudomonas aeruginosa	4.5e-46
BLP4_PSEAI	Beta-lactamase PSE-4	Pseudomonas aeruginosa	9.2e-46
BLP1_PSEAI	Beta-lactamase PSE-1	Pseudomonas aeruginosa	1.6e-45
BLAC_RHOCA	Beta-lactamase	Rhodobacter capsulatus	1.0e-43
BLAC_STAAU	Beta-lactamase	Staphylococcus aureus	1.1e-43
BLC4_PSEAI	Beta-lactamase CARB-4	Pseudomonas aeruginosa	1.7e-43
BLC6_VIBCL	Beta-lactamase CARB-6	Vibrio cholerae	3.2e-43
BLA1_MORCA	Beta-lactamase BRO-1	Moraxella catarrhalis	1.7e-30
BLE1_PSEAI	Extended-spectrum beta-lactamase PER-1	Pseudomonas aeruginosa	1.8e-20
BLAC_BACUN	Beta-lactamase	Bacteroides uniformis	7.4e-15
BLAC_BACVU	Beta-lactamase	Bacteroides vulgatus	1.7e-09

Supplementary Figure S2: Table generated by HMMER web server (version 1.8). The first column lists SwissProt codes of the sequences used for the alignment. For each SwissProt code a description of the protein name and/or family, organism's species and E-value have been associated for clarity and sake of completeness.

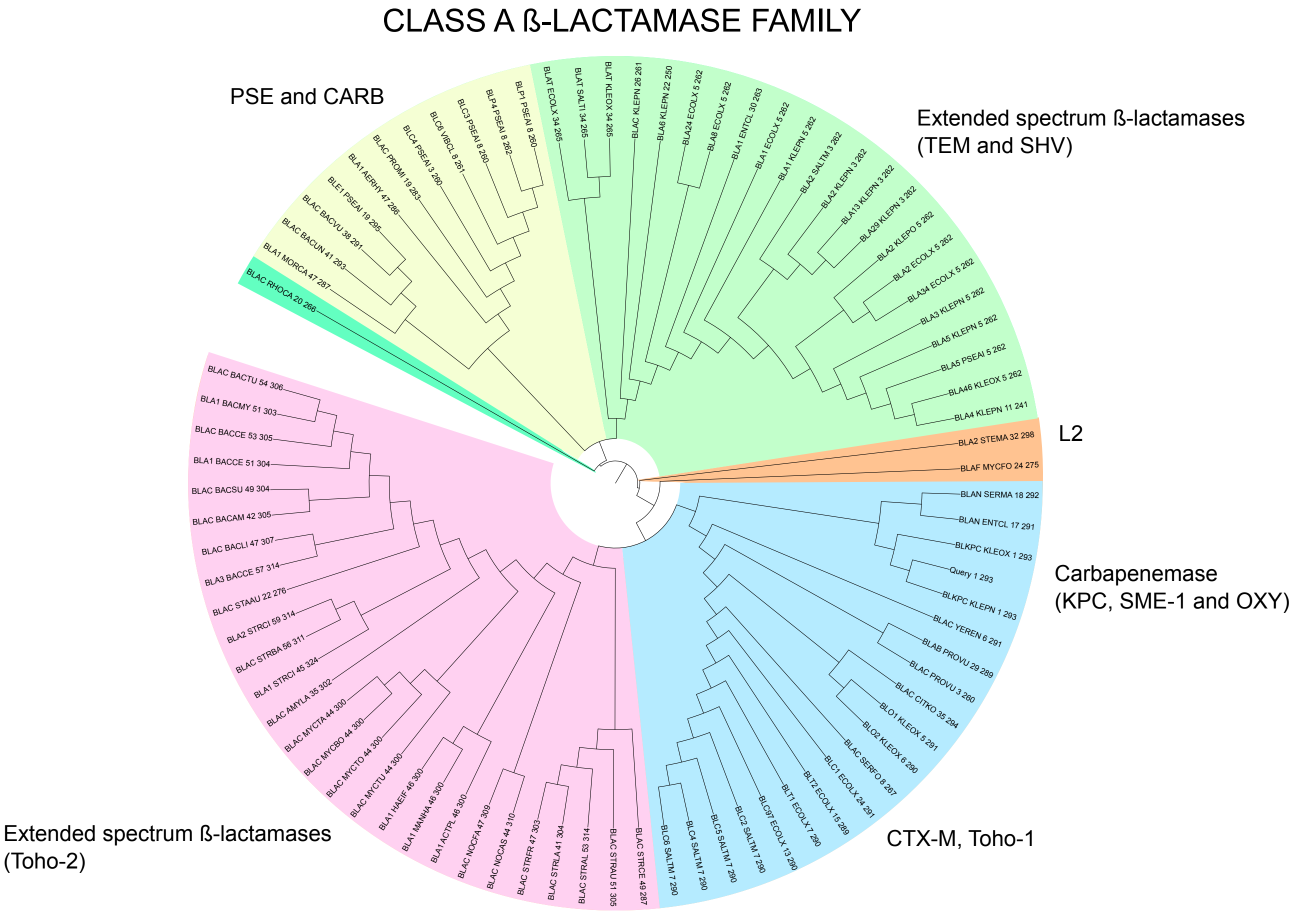

Supplementary Figure S3: Evolutionary tree of the sequences used in the alignment. Major families have been identified with different colours. All sequences belong to the class A β-lactamase family.

System	Total Simulation Time (μs)	
	Unbiased Simulations	PT-metadynamics Simulations
WT	18.5	2.4
$A^{77}N$	8.0	2.4
$L^{102}T$	8.0	2.4
$I^{108}N$	8.0	2.4
dMUT	8.0	2.4
$L^{138}N$	8.0	2.4
$L^{199}R$	8.0	2.4
SPR-3-226	8.0	2.4

Table S1: Summary of simulations. The reported time corresponds to the accumulated simulation time of all the non-adaptive and adaptive simulations (unbiased simulations), and of all the replicas of the PT-metaD simulations (biased simulations).

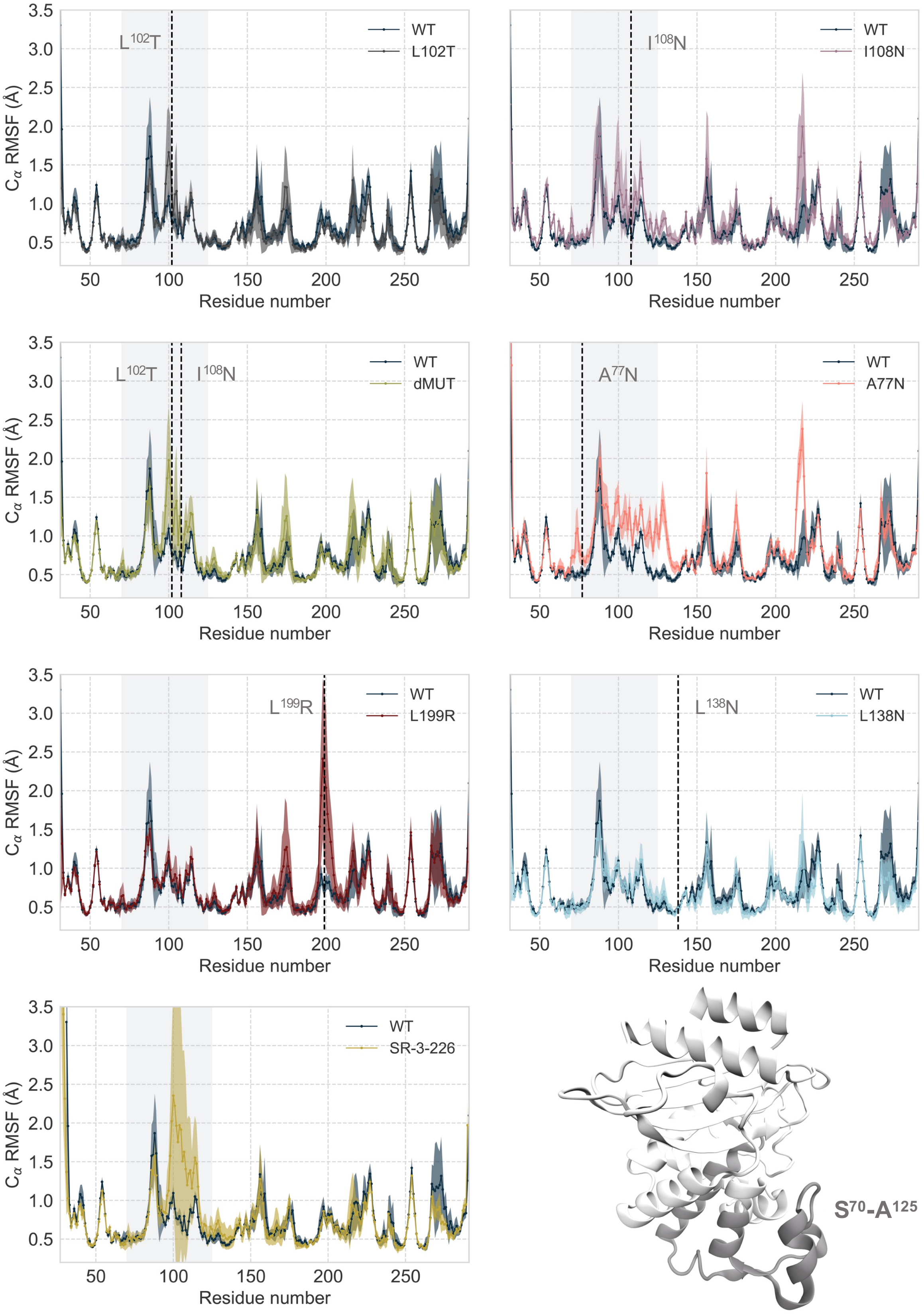

Supplementary Figure S4: RMSF profiles of KPC-2 WT, mutants and ligand bound structures derived from the whole set of the unbiased simulations. Mean values are represented as lines and standard deviation is indicated by shading. The most flexible region S^{70}-A^{125} with respect to the WT in most of the systems has been highlighted in the graphs and represented in grey in the crystal structure.

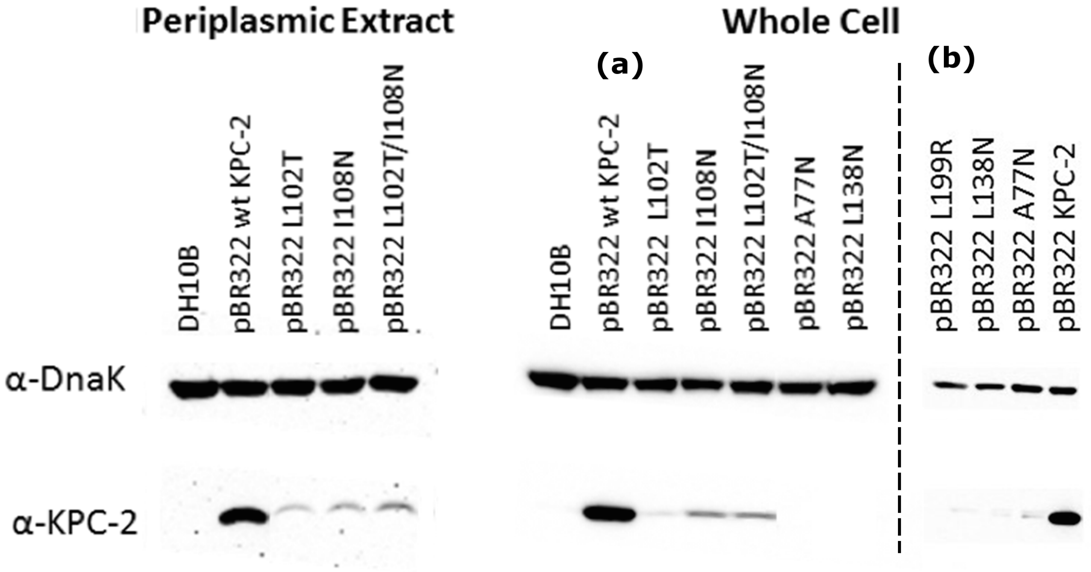

Figure S5: Immunoblots of periplasmic extract and whole cell lysate of DH10B cells expressing pBR322-catI-blaKPC-2 wild-type or variants ($A^{77}N$, $L^{102}T$, $I^{108}N$, $L^{138}N$, $L^{199}R$ or $L^{102}T/I^{108}N$). DnaK is a loading control. The whole cell lysate extract was run in two separate gels demarcated by the dashed lines and marked a and b).

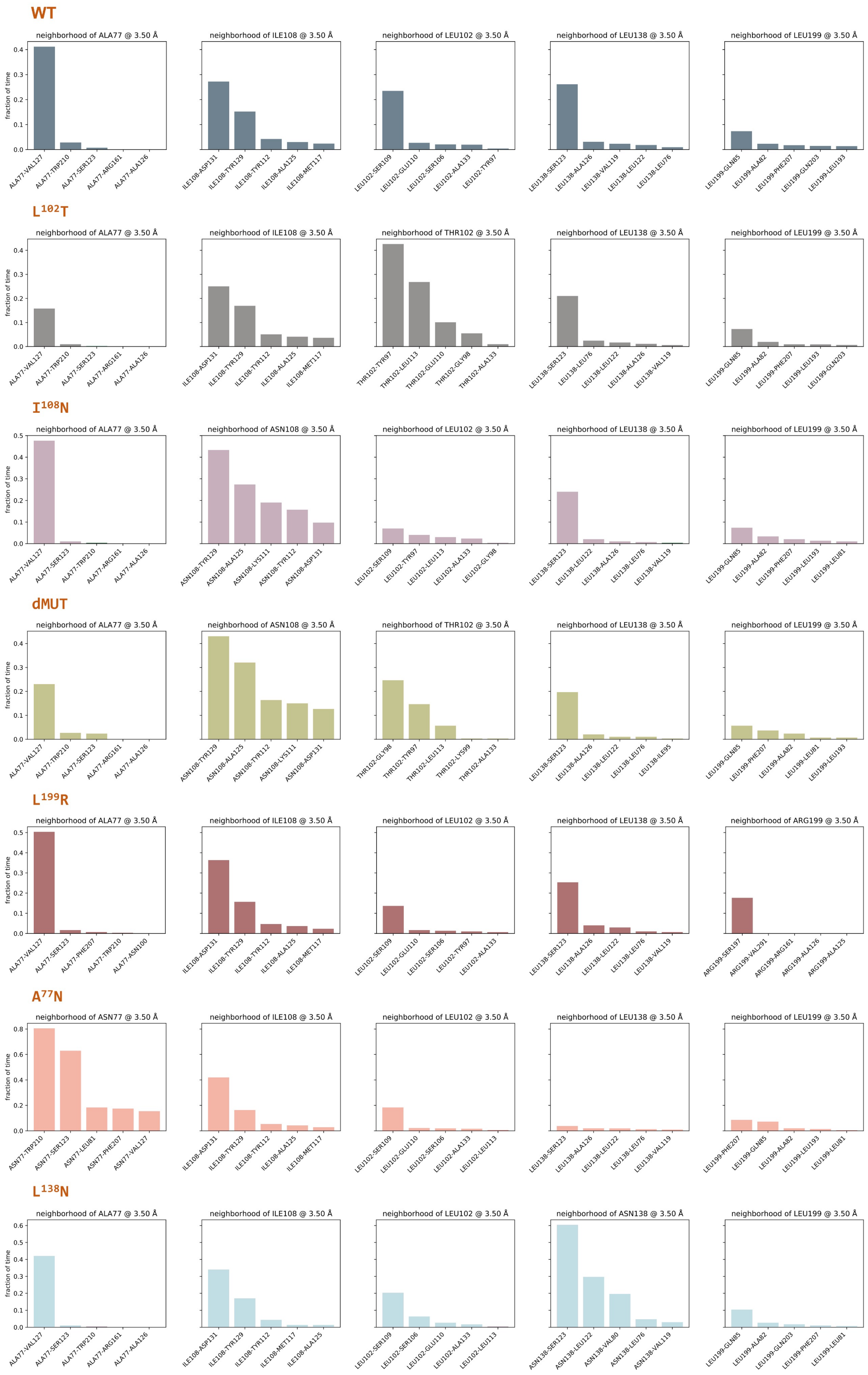

Figure S6: Important interactions formed between the mutated/WT residues and neighbour residues over the course of the unbiased simulations. As neighbour residues were considered the ones that their heavy atoms were 3.50 Å away from the mutated residue.

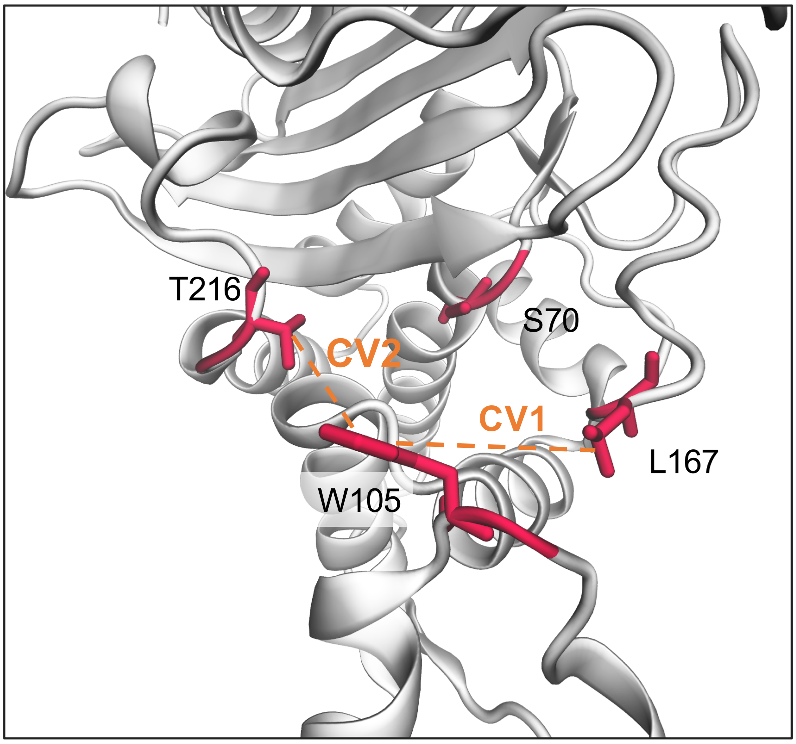

Supplementary Figure S7: Collective variables used in metadynamics simulations. CV1 describes the distance between the centre of mass of the indole ring of the side chain of W^{105} and the C_β of L^{167}. CV2 describes the distance between the centre of mass of the indole ring of the side chain of W^{105} and the C_β of T^{216}.

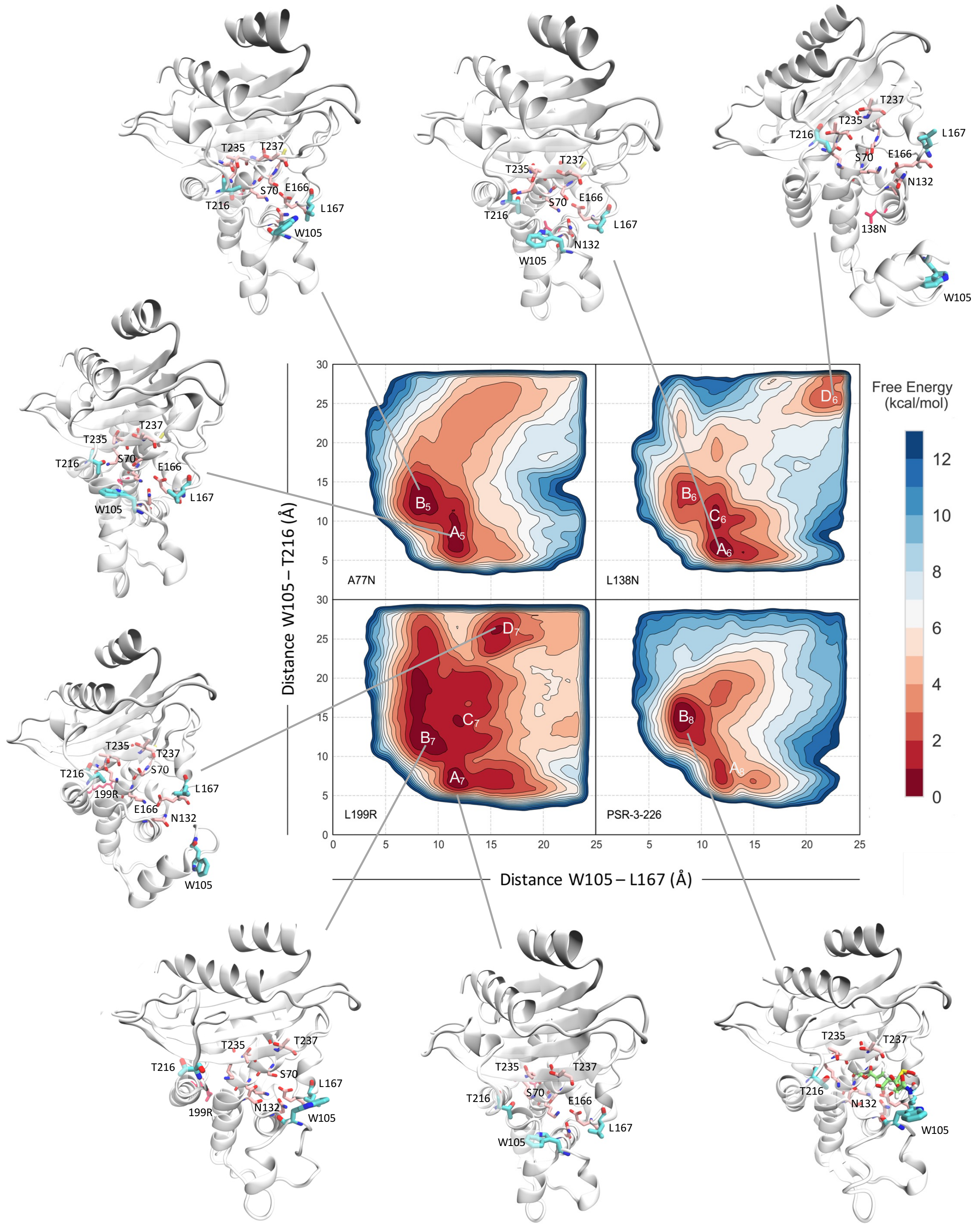

Figure S8: Free-energy surfaces (FES) plots of the KPC-2 single mutants $A^{77}N$, $L^{138}N$, $L^{199}R$ and the KPC-2 bound to PSR-3-226. The FES have been reconstructed along the two variables, CV1 (distance between the center of mass of the indole ring of the side chain of W^{105} and C_β of L^{167}) and CV2 (distance between the center of mass of the indole ring of the side chain of W^{105} and C_β of T^{216}). Structures extracted from their corresponding basins have been illustrated. The mutated residues are shown in red sticks, while residues that have been reported to be important for the catalysis [] are shown in pink sticks.

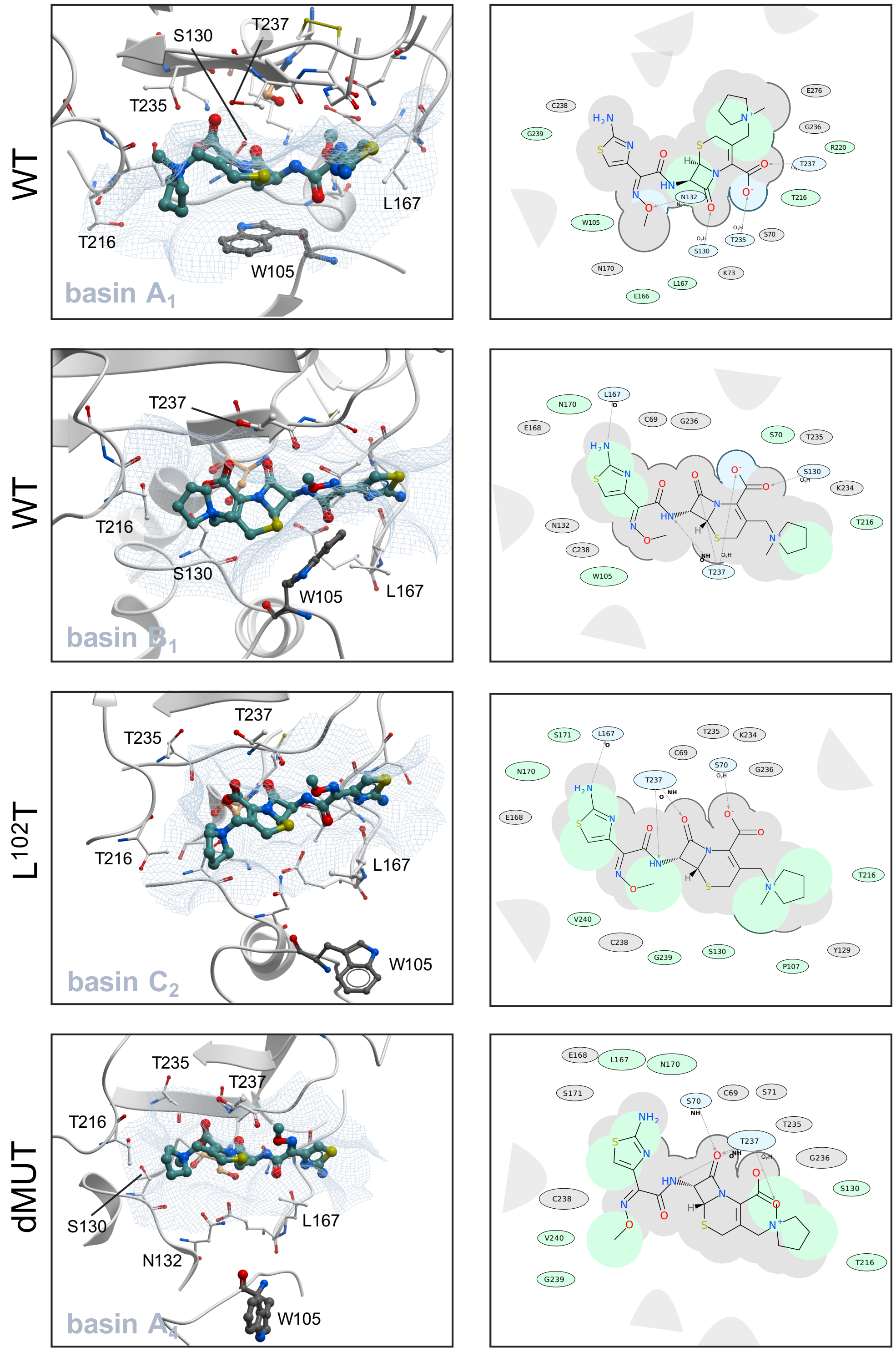

Figure S9: Binding poses and ligand interaction diagrams resulting from the docking of the experimentally tested antibiotic cefepime to exemplar conformations corresponding to selected basins of the free energy surfaces (Figure 3, main text). Coloring explanation of the ligand interaction diagrams: green shading represents hydrophobic regions, blue shading represents hydrogen bond acceptor, white dashed arrows represents hydrogen bonds, grey parabolas represents accessible surface for large areas, broken thick line around ligand shape indicates accessible surface, while the size of residue ellipse represents the strength of the contact.

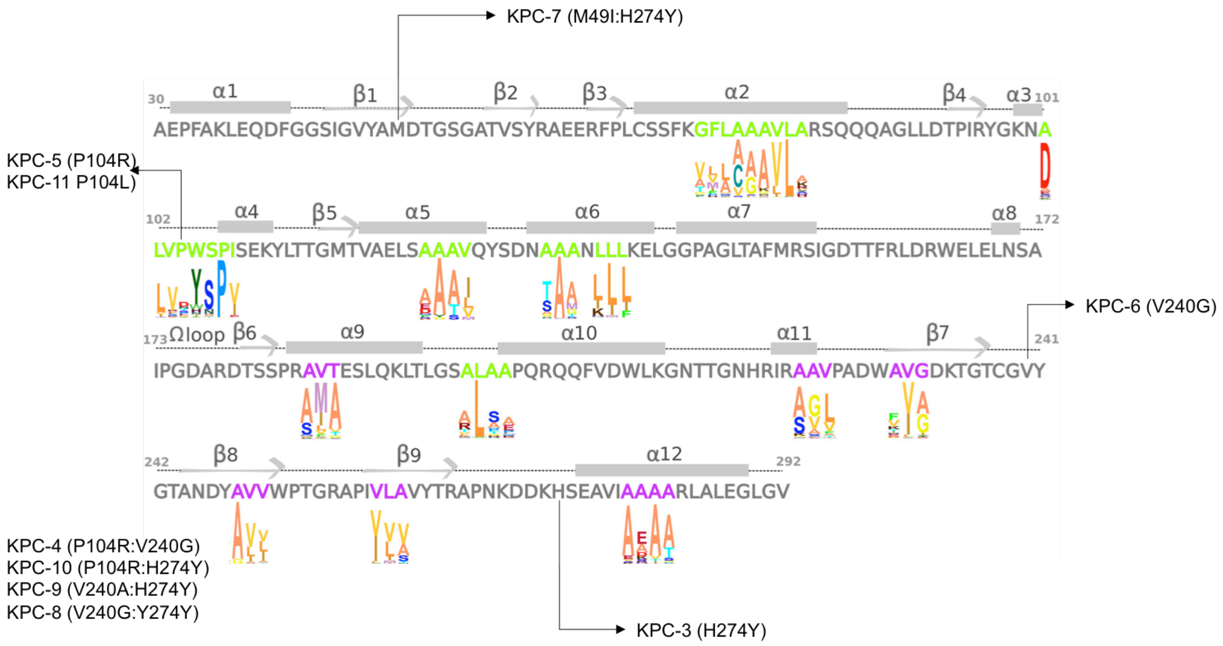

Figure S10: Sequence comparison of KPC variants. The difference between KPC2 and KPC3 is the H247Y mutation. There is complete conservation within the core and the hydrophobic nodes between the two variants.
